# Multi-scale stochastic organization-oriented coarse-graining exemplified on the human mitotic checkpoint

**DOI:** 10.1038/s41598-019-40648-w

**Published:** 2019-03-07

**Authors:** Richard Henze, Chunyan Mu, Mate Puljiz, Nishanthan Kamaleson, Jan Huwald, John Haslegrave, Pietro Speroni di Fenizio, David Parker, Christopher Good, Jonathan E. Rowe, Bashar Ibrahim, Peter Dittrich

**Affiliations:** 1 Faculty of Mathematics and Computer Science, Friedrich Schiller University Jena, Jena, Germany; 2 School of Computing, Teesside University, Teesside, UK; 3 Faculty of Electrical Engineering and Computing, University of Zagreb, Zagreb, Croatia; 4 School of Computer Science, University of Birmingham, Birmingham, UK; 5 School of Mathematics, University of Birmingham, Birmingham, UK; 6 Mathematics Institute, University of Warwick, Warwick, UK; 7 Chair of Bioinformatics, Matthias Schleiden Institute, Friedrich Schiller University of Jena, Jena, Germany

## Abstract

The complexity of biological models makes methods for their analysis and understanding highly desirable. Here, we demonstrate the orchestration of various novel coarse-graining methods by applying them to the mitotic spindle assembly checkpoint. We begin with a detailed fine-grained spatial model in which individual molecules are simulated moving and reacting in a three-dimensional space. A sequence of manual and automatic coarse-grainings finally leads to the coarsest deterministic and stochastic models containing only four molecular species and four states for each kinetochore, respectively. We are able to relate each more coarse-grained level to a finer one, which allows us to relate model parameters between coarse-grainings and which provides a more precise meaning for the elements of the more abstract models. Furthermore, we discuss how organizational coarse-graining can be applied to spatial dynamics by showing spatial organizations during mitotic checkpoint inactivation. We demonstrate how these models lead to insights if the model has different “meaningful” behaviors that differ in the set of (molecular) species. We conclude that understanding, modeling and analyzing complex bio-molecular systems can greatly benefit from a set of coarse-graining methods that, ideally, can be automatically applied and that allow the different levels of abstraction to be related.

## Introduction

Biological processes like cell-cycle control^1^ are complex due to the number of components involved and due to non-linearity and ubiquitous feed-back loops^2^. There is usually a trade-off between the accuracy of a model, desirable for representing biological knowledge in detail^3^, and the simplicity of a model, which is beneficial for understanding and generalizing the fundamental mechanisms involved, e.g.^4,5^. If both are required, a multi-model approach is useful, where a set of models with different granularity is derived. A model can be created by the refinement of a simpler one, or by the REVcoarse-graining^6–8^ of a detailed model (the approach followed in this paper). A central problem of this approach however is how to align the different models in a coherent way^9^.

**Figure 1:**
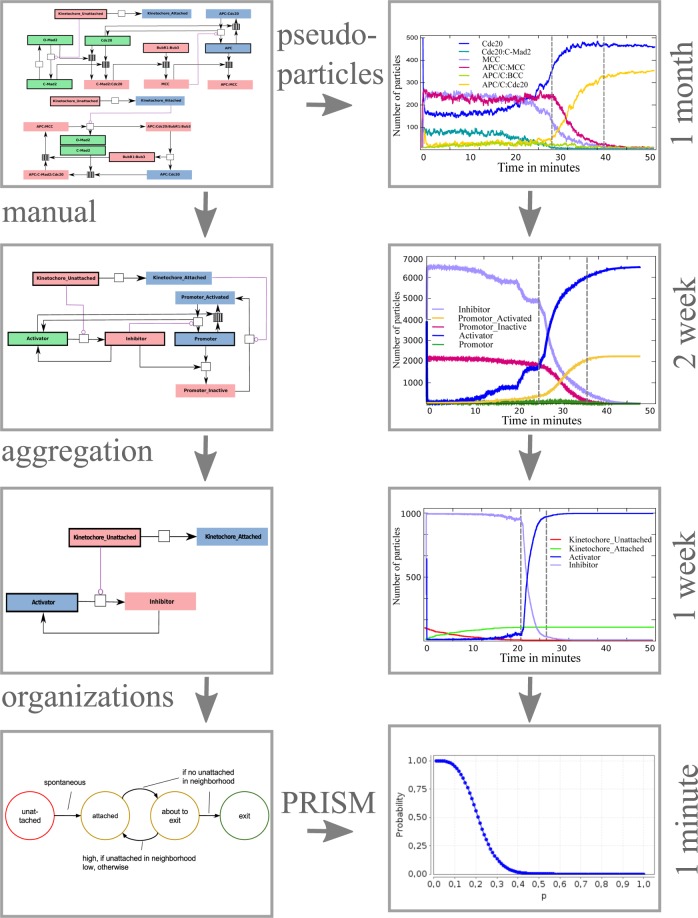
Overview of the four models (top to bottom: full model, reduced model, coarsest model and compartmentalized model) and the coarse-graining methods used to derive them (manual, aggregation and organizations; see Sec. 2.1). Left: The frameworks of all models, with the corresponding techniques used to derive them from the previous model. Right: The simulation results from the corresponding models, including the single CPU simulation time.

Here, we demonstrate how different coarse-graining methods can help in dealing with the complexity of biological molecular systems. The basic idea is to look at the inherently non-linear dynamical system from different levels of abstraction that are related to each other and thus form a hierarchy of coarse-grainings (Fig. 1).

We combine formal methods that are based on strict mathematical principles with manual coarse-graining, which allows us to include domain expert knowledge that is difficult if not impossible to formalize. We will also see that in practice it appears beneficial to depart from the strict mathematical definition of a coarse-graining in favor of obtaining a more elegant (e.g. less detailed) model through an approximate coarse-graining, as we will do by using organizational coarse-graining based on chemical organization theory^10^.

We will do so by studying the (mitotic) spindle assembly checkpoint^11,12^. To guarantee genomic integrity and viability, the cell must ensure proper distribution of the replicated chromosomes among the two daughter cells in mitosis. The mitotic spindle assembly checkpoint (SAC) is a central regulatory mechanism to achieve this goal. The SAC is an evolutionary conserved mechanism, exclusively sensitive to the state of kinetochores attached to microtubules. One single unattached kinetochore is sufficient to withhold mitotic progression. A dysfunction of this checkpoint may lead to aneuploidy and likely contributes to the development of cancer^13,14^. Kinetochores of unattached or misaligned chromosomes generate a diffusible “wait-anaphase” signal, which is the basis for downstream events to inhibit the anaphase promoting complex/cyclosome (APC/C or APC)^15–17^. During metaphase the APC stays inhibited and quickly switches to its active form after the final proper spindle attachment. Activated APC cleaves the sister chromatid and initiates anaphase.

The methods we are applying here can be seen as an addition to the large body of established coarse-graining methods^18–20^. These methods can be roughly grouped into those that are independent of the reaction network structure, like quasi steady state approximation or computational singular perturbation^21,22^, and those that take the reaction network structure into account, such as tropical analysis^23^, limiting step inspired methods^24^, and rule-based fragmentation^8^. Our approach, also taking reaction network structure into account, differs from the conventional coarse-graining techniques in the following way: We add domain knowledge when reducing the dimensions and semantically annotate the coarser species. We are also switching between model classes, that is, from stochastic particle simulation, over deterministic continuous differential equations, stochastic discrete dynamics, temporal model checking, and a stochastic discrete spatial model. Finally, we link the different models by chemical organizations across model class borders.

In the next section we will briefly review the methods and hierarchy of models. Subsequently, we will present the results, starting with the most detailed model of the human mitotic checkpoint and showing how a series of coarser models can be derived, while keeping the essential checkpoint behavior intact.

## Methods and Mathematical Framework

This work shows how to orchestrate different methods published elsewhere in order to obtain a set of models (Table 1) with varying granularity that are clearly linked to each other. Below, we give overviews of the methodological procedure and of the hierarchy of models.

**Table 1:** Overview of the models.

Models Name	Description	Source
Full SAC Model (Model 1)	14 species, 21 reactions, [Model 1b has 19 reactions].	^25^
Reduced SAC Model (Model 2)	7 species, 9 reactions, [Model 2b has 8 reactions].	Manual lumping of species from Model 1
Coarsest SAC Model (Model 3)	4 species, 3 reactions, [Model 3b has 2 reactions].	Aggregation analysis of Model 1
Discrete 4-state Model (Model 4)	4 states, 4 state-transitions	Organization analysis of Model 3

### Brief Overview of the Methods

1. We start with a detailed reaction network model^25^ and its 3D particle-based dynamical simulation^26^. This detailed model will be coarse-grained in various ways, manually and automatically. The model is derived and described in detail in the literature^25^.
2. Manual coarse-graining is done with respect to the desired function, e.g., checkpoint behavior (Fig. 1). Automatic coarse-graining is performed by:

- organization-oriented techniques^27^ including discrete organizations^28^ and spatial organizations^29,30^; and by
- aggregation lattice methods^31^

3. From the coarse-graining, we derive a minimal stochastic model (with a small number of states but including space) with a state space such that exact analysis via probabilistic model checker PRISM^32^ is feasible. Note that model checking may include further automatic coarse-graining of states, for example through bisimulation or finite-horizon bisimulation^33^. However, this coarse-graining is internal to the model checking tools and hidden for the user; it is thus not discussed further in this work.
4. We coarse-grain space by compartments, obtaining a compartmentalized model, where each compartment is a well-stirred reaction vessel whose state is defined by a (discrete) concentration vector or by a small set of (four) states. Compartments are connected by diffusion according to a graph structure. In a stochastic 4-state model, diffusion is modeled by letting the state transition probability of a compartment (modeling a kinetochore) depend on the states of neighboring compartments. Note that in this paper we will not simulate a compartmentalized ODE model; it is only used conceptually for discussing spatial organizations.

The methods are explained in more detail below (see also (Table 2)).

**Table 2:** Overview of the approaches.

Approach	Description
Spatial stochastic simulation	The method uses a reaction network to simulate reacting molecules within a sphere like reactor, mimicking a human cell, using ReaDDy (see Sec. S1.1)^26^. Kinetochores are spread randomly over a plate located in the middle of the cell. Reaction rates are defined according to the rates specified by the reaction network model. Sizes and diffusion coefficients of the species are taken from the literature and calculated, respectively (see Tables 3, 5 and 6).
Spatial state-transition simulation	This method uses time-discrete state-updates. A set of kinetochores with discrete states (here, four states) is simulated by placing them randomly over a plate. A spatial neighborhood relation between kinetochores influences their state transition (see Sec. S1.2).
Chemical organizations and probabilistic model checking	Chemical reaction networks are modeled as continuous-time Markov chains. Probabilistic model checking is used to compute exact state-transition probabilities and other stochastic quantities. Chemical organizations are identified by model decompositions and a quantitative dynamical analysis in terms of the identified organizations is performed by applying probabilistic model checking. As a result, a coarse grained Markov chain model of hierarchical organizations for the reaction network is constructed (see Sec. S1.3 and S1.4).
Approximate aggregation	An ODE model is reduced based on quadratic matrix approximation (see Supplement).

### Brief overview of the Models

Three different network models (Model 1 to Model 3) are considered, as well as a state transition model (Model 4). Model 1^25^ is the starting point from which the other models are derived by different coarse-grainings. Dynamical models relying on the reaction network models. A reaction network has to be combined with a model of the dynamics in order to simulate dynamical behavior. The approaches we consider are spatial stochastic particle simulation (ReaDDy^26^), quasi-spatial state-transition simulation, probabilistic model checking (PRISM^32^) and ordinary differential equations (see Table 2).

### Stochastic simulation with ReaDDy

Detailed spatial particle simulations were performed using the efficient simulation framework ReaDDy^26^. Every species is modeled as a sphere for the sake of simplicity. Assuming a homogeneous density of protein, its sphere’s radius can be derived from its mass, which is often known. The reactor size is chosen according to the size of the cell nucleus. In our setup, all particles are forced to stay in the kinetochore region in the middle of the nuclear space. The initial concentration of all species is translated into a number of particles, via the reaction volume. All particles, including 92 kinetochores, are placed randomly in the reaction vessel and undergo a Brownian motion.

Reactions can be defined in a separate file, including their reaction rates. Once two particles enter their common reaction space and a reaction is defined between them, the rate determines randomly if the reaction is carried out. First order reactions, like the spontaneous attachment of kinetochores, happen purely at random based on the rate.

Furthermore, we introduced pseudo-particles, as regarding concentration refer to millions of particles, which would be impossible to simulate in sufficient time. One of our pseudo-particles represents 1000 real particles. We simulated those particles with realistic diffusion coefficients for a coarse grained time, corresponding to 20 real time minutes.

### Quasi-Spatial State-Transition Simulation of the 4-State Model

To simulate the 4-state model we define a kinetochore as a point on a two-dimensional Cartesian-coordinate-system. 92 kinetochores are placed on a plane randomly. Their initial state is ‘unattached’, which switches spontaneous to ‘attached’ (probability 0.005 per time step). The parameter *p* ∈ [0, 1] is the probability (per time step) of the transition from state ‘attached’ to state ‘about to exit’. Every time step all nodes can change spontaneously to the ‘attached state’ and further to the ‘about to exit’ state. A node in state ‘about to exit’ checks whether all of its neighbors are also in the ‘about to exit’ state or ‘exit’ state; if this is true, the node irreversibly changes to ‘exit’, otherwise it falls back to the ‘attached’ state.

Neighbors are nodes that are positioned within a certain range.

Python source code is freely available.

### Chemical Organization Theory (COT)

#### Reaction network

A *reaction network* $\langle  {\mathcal M} , {\mathcal R} \rangle$ is defined by a set of molecular species ${\mathcal M}$ and a set of reactions ${\mathcal R}$ occurring among the molecular species ${\mathcal M}$. For each reaction $r\in  {\mathcal R}$, let LHS(*r*) and RHS(*r*) denote the set of reacting and produced species of reaction *r*, respectively. A reaction network’s stoichiometric matrix *N* = (*n*_*i*,*r*_) is an $| {\mathcal M} |\times | {\mathcal R} |$ matrix of stoichiometric coefficients *n*_*i*,*r*_, where *n*_*i*,*r*_ denotes the net amount of molecules of species $i\in  {\mathcal M}$ produced by reaction $r\in  {\mathcal R}$. For example, for the reaction *r*: KinU + O-Mad2 → KinU + C-Mad2, *n*_KinU,*r*_ = 0, *n*_O -Mad2,*r*_ = −1 and *n*_C -Mad2,*r*_ = 1.

Given a set of species $A\subseteq  {\mathcal M}$, we define ${ {\mathcal R} }_{A}=\{r\in  {\mathcal R} |{\rm{LHS}}(r)\subseteq A\}$ as the set of reactions that can “fire” in *A* and we define ${\rm{dp}}(A)={\cup }_{r\in { {\mathcal R} }_{A}}\,{\rm{RHS}}(r)$ as the set of species that can be *directly produced* by the reactions that can fire in *A* (see ref.^34^ for relation to point set topology, where dp(*A*) it is denoted by *cl(A*)).

#### Closure

A subset of molecular species $A\subseteq  {\mathcal M}$ is *closed*, if ${\rm{dp}}(A)\subseteq A$, that is, if the application of all possible reactions from ${\mathcal R}$ on *A* only produces species from *A*^35,36^. For each set of species $B\subseteq  {\mathcal M}$ there exists a unique smallest closed set *G*_*CL*_(*B*) containing *B*, *B* ⊆ *G*_*CL*_(*B*)^10,37^. We say that *G*_*CL*_(*B*) is the *closure* of *B*. Intuitively, the closure of a set of species contains these species and all those species that can be reached by an arbitrary long reaction path starting with species from *B*^35^. From an algorithmic perspective, we can construct the closure iteratively by *G*_*CL*_(*B*) = *B* ∪ dp(*B*) ∪ dp(dp(*B*)) ∪ …, or recursively by *B*_0_ := *B*, *B*_*i*+1_ : = *B*_*i*_ ∪ dp(*B*_*i*_) with *G*_*CL*_(*B*) = *B*_∞_ = lim_*i*→∞_ *B*_*i*_ ^36^.

#### Self-maintenance

Let *N* be the stoichiometric matrix of the reaction network, a set of molecular species $A\subseteq  {\mathcal M}$ is self-maintaining, if there exist a flux vector $v=({v}_{r}),{v}_{r}\in {\mathbb{R}}$, such that *v*_*r*_ > 0 for $r\in { {\mathcal R} }_{A}$, *v*_*r*_ = 0 for $r\notin { {\mathcal R} }_{S}$, and *Nv* ≥ 0^10^. That is, for a self-maintaining set of species we can find a strictly positive rate for each reaction that can fire in that set (while all other reactions have rate of zero), such that no species decays. In consistent reaction networks^10^, for each set of species $B\subseteq  {\mathcal M}$ there exists a unique largest self-maintaining set *G*_*SM*_(*B*) subset of *B*, *G*_*SM*_(*B*) ⊆ *B*. We say that *G*_*SM*_(*B*) is the self-maintaining set generated by *B*. Intuitively, we can obtain this self-maintaining set by successively removing those species that are not sufficiently produced.

#### Organization

A set of species $A\in  {\mathcal M}$ that is closed and self-maintaining is called an organization^10,36^. In consistent systems, for each set of species $B\subseteq  {\mathcal M}$ we can uniquely generate an organization *G*_*O*_(*B*) = *G*_*SM*_(*G*_*CL*_(*B*)), that is we first generate the closure of *B* by adding to it all possible molecules that can be produced. For the resulting closed set, we generate the self-maintaining set by removing all those molecules that are not produced. The resulting organization is the largest set of species that might be able to coexist in a stable way, when starting from an initial state with species from *B*. Thus, organizations are related to the dynamical behavior implied by the reaction network. Given a reaction network and a kinetic law like mass action kinetics, it has been proven that the fixed points of the obtained ordinary differential equation (ODE) system, relate to the set of organizations as follows. For every fixed point, the set of molecules with strictly positive concentrations in that fixed point is an organization of the reaction network^10^. The same is true for periodic attractors and many other limit sets^38^.

Procedure for the organizational analysis:

1. Decide the temporal (and spatial) scale and thus the reactions to be considered. Here, we consider two situations:
  **Situation “before attachment”** (**Pro-Metaphase**), without reaction kinetochore attachment, i.e., assuming unattached kinetochores and assuming a short time scale in which no attachments take place;
  **Situation “attachments”** (**Anaphase**), including attachment of kinetochores, i.e., long time scale (next section)
2. Calculate all organizations and plot their Hasse diagram.
3. Check criteria for those **organizations being interesting**. For example, here we require that an “interesting” organization contains at least one kinetochore, being attached or not, which translates into the constraint “[KinU] + [KinA] > 0” or “(KinU OR KinA)” for short.
4. The resulting hierarchy of organizations can be used to explain the dynamics by mapping states to organizations and to guide further coarse grainings, by trying to reduce the number of organizations while keeping the “interesting” ones.

### Stochastic simulation and temporal model checking with PRISM

In this section, we review techniques^32^ for formal quantitative analysis of chemical reaction networks, using discrete stochastic models represented as continuous-time Markov chains and probabilistic model checking. We present methods to automatically identify organizations, and to study quantitative properties regarding movements between these organizations. A coarse grained Markov chain model of hierarchic organizations for a given reaction network is then constructed. The organization-based coarse grained model can then be used to approximate and predict the behavior of the original reaction network. We have implemented the techniques as an extension of the probabilistic model checking tool PRISM^39^.

We model the dynamics of a reaction network as a continuous-time Markov chain (CTMC). The formal definition is presented in Definition 1. A *state* here is defined by a discrete number for each molecular species.

#### **Definition 1**

(**CTMC for reaction network**) *Given a reaction network* $\langle  {\mathcal M} , {\mathcal R} \rangle$, *a volume limit* ${N}_{{\max }}\in {\mathbb{N}}$ *and a rate function* ${{\mathtt{rate}}}_{r}\,:{{\mathbb{N}}}^{ {\mathcal M} }\to {{\mathbb{R}}}_{\ge 0}$ *for each* $r\in  {\mathcal R}$, *we define the corresponding CTMC* **A** = (*Q*, *Q*_0_, Δ, *L*) *where*:

- $$Q=\{q: {\mathcal M} \to {\mathbb{N}}|{\sum }_{s\in  {\mathcal M} }\,q(s)\le {N}_{{\max }}\}$$

*is the set of population counts of* ${\mathcal M}$ *and* Δ *is defined as follows*. *For states q*, *q*′ ∈ *Q*, *we write* $q\,\mathop{\longrightarrow }\limits^{(R,P)}\,q^{\prime}$ *if and only if, for each species* $s\in  {\mathcal M}$, *we have q*(*s*) ≥ *R*(*s*) *and q*′(*s*) = *q*(*s*) − *R*(*s*) + *P*(*s*), *and* ${\sum }_{s\in  {\mathcal M} }\,q^{\prime} (s)\le {N}_{{\max }}$. (*R*, *P*) = *r denotes a reaction* $r\in  {\mathcal R}$ *with reactands R and products P*. *The stoichiometric factors R*(*s*) *and P*(*s*) *denote the number of molecules of species s consumed and produced by the reaction r*, *respectively. Then, for any q*, *q*′ ∈ *Q*, *we have*:

- ${\rm{\Delta }}(q,q^{\prime} )=\sum \,\{{{\mathtt{rate}}}_{r}(q)|r\in  {\mathcal R} \,and\,q\,\mathop{\longrightarrow }\limits^{r}\,q^{\prime} \}$, *and we call r the transition label of* $q\,\mathop{\longrightarrow }\limits^{r}\,q^{\prime}$.

*Q*_0_ *can be any subset of Q representing initial configurations of interest*, *and L can be any labeling function over Q that identifies states with relevant properties*.

The transition rate matrix Δ assigns a rate to each pair of states in the CTMC, which is used as a parameter of a distribution. Here, we follow the general law of mass-action by setting ${{\mathtt{rate}}}_{r}(q)={\lambda }_{r}\cdot {\prod }_{s\in {\rm{LHS}}(()r)}\,q(s)$ with *λ*_*r*_ being a kinetic rate constant for reaction *r* (and assuming the stoichiometric coefficient of each reactant is at most one).

For instance, Fig. S1 presents the stochastic state transition diagram for the coarsest SAC Model in pro-meta phase (short time scale) with ${N}_{{\mathtt{\max }}}=5$.

With a limited total amount of molecules, both cases of too few and too many molecules can prevent reaction rules being fired. As a consequence, we need to define *discrete organizations*, and the states contributing to generate them. Given a state *q*, ${ {\mathcal R} }_{q}$ denotes the reactions firing in any of the reachable states of *q*.

#### **Definition 2**

(**Discrete organization and internal generator**^28^) *Let* $( {\mathcal M} , {\mathcal R} )$ *be a reaction network*. *A subset of species* $D\subseteq  {\mathcal M}$ *is called a discrete organization if there is a state q* ∈ *Q such that*: (*i*) *ϕ*(*Acc*(*q*)) = *D* (*closure*); *and* (*ii*) *there is sequence of transition labels* (*r*_1_, …, *r*_*k*_ *where* ${r}_{i}\in  {\mathcal R}$ *such that* ${\cup }_{i=1}^{k}\{{r}_{i}\}={ {\mathcal R} }_{q}$ *and* $q^{\prime} =({r}_{k}\circ \ldots \circ {r}_{1})(q)$ *satisfies* $\forall \,s\in D:q^{\prime} (s)\ge q(s)$ (*self-maintenance*). *Such a state q is called an internal generator of the discrete organization*.

*ϕ*(*q*) denotes the set of molecular species that are present, i.e. *ϕ*(*q*) = {*s*|*q*(*s*) > 0}. *Acc*(*q*) ⊆ *Q* denotes the set of states that are reachable (“accessible”) from *q*. Thus, $\varphi (Acc(q))={\cup }_{q\in Acc(q)}\varphi (q^{\prime} )$ is the set of all species that can be produced in an arbitrarily long sequence of reactions when starting from state *q* (including the species of *q*). ${ {\mathcal R} }_{q}$ is the set of all reactions that eventually can fire when starting with state *q*.

#### **Definition 3**

(**Generator**) *A state q*′ ∈ *Q is called a generator of organization D iff* $\exists \,q\in Acc(q^{\prime} )$ *such that q is an internal generator of D*.

Note that, in general, the organization *D* generated by a state *q*′ is not unique. However, if *q* is an internal generator, there is only one organization it generates.

In order to analyze the system behavior and perform an organization-based quantitative analysis of the reaction network, we study the connections between chemical organizations and the decompositions into strongly connected components (SCCs) of the Markov chain.

#### **Definition 4**

(**SCC**^40^) *A strongly connected component* (*SCC*) *of a Markov chain is a maximal set of states T such that*, *for every pair of states q* and *q*′, *there is a path from q to q*′.

Intuitively, in the Markov chain for a reaction network, SCCs are important for an organization-based analysis. However, some but not all SCCs correspond to organizations. We first note that *bottom* strongly connected components do relate to organizations.

#### **Definition 5**

(**BSCC**) *A bottom strongly connected component* (*BSCC*) *is an SCC T from which no state outside T* is reachable from *T*.

#### **Proposition 1**

*Each BSCC corresponds to a* (*unique*) *organization*, *which is generated* (*uniquely*) *by any state of that BSCC*.

However, there are organizations whose internal generators are *not* contained in any BSCC. In order to also include such organizations, we call SCCs that correspond to an organization *good SCCs*.

#### **Definition 6**

(**Good SCC**) *An SCC T is called good if it contains a cycle of the firing of every “possible” reaction rule*, *i*.*e*., *those whose reactants R appear in the SCC* ($R\subseteq \{\varphi (q)\mathrm{\ |\ }q\in T\}$).

Clearly, some generators can contribute to multiple organizations. This makes it more difficult to decompose the Markov model into its sets of generators. However, internal generators located in good SCCs contribute uniquely to an organization.

#### **Proposition 2**

*A generator g is an internal generator of organization D if it is located in a good SCC T such that:* $g\in T\wedge {\cup }_{q\in T}\,\varphi (q)=D$.

#### **Proposition 3**

*Given a good SCC T*, *let A* = *ϕ*(*T*), *if A is closed, then A is a discrete organization and T is the set of internal generators of A*.

We compute the (discrete) organizations of a reaction network by analyzing the strongly-connected components of its Markov chain’s underlying transition graph. Since every state in a good SCC is an internal generator of an organization, we identify good SCCs to find organizations. We then use probabilistic model checking to analyze quantitative properties regarding the dynamics of the network with respect to its organizations. Such organization-based quantitative analyses can be used to construct the structure of organization-based coarse-grained model, and provide a framework to approximate the complex dynamical behaviors of the original reaction networks.

## Results and Discussions

### Full SAC Model (Model 1)

**Figure 2:**
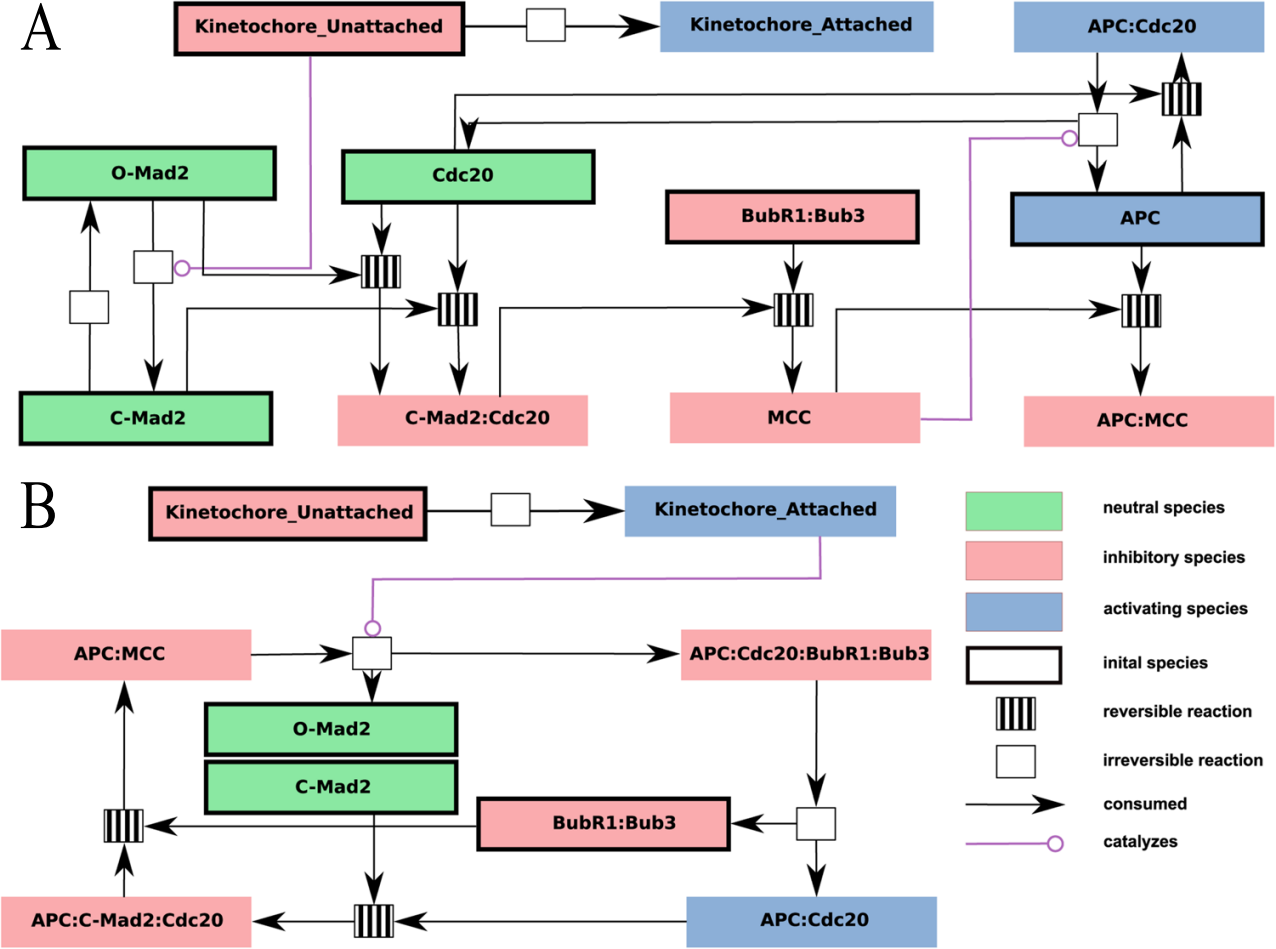
Reaction network of the complete SAC Model (Model 1) depicted for two situations (activated (Panel A) and silenced checkpoint (Panel B). The meaning of symbols and colors is explained in the legend in the bottom right corner. This model is based on the work by Henze *et al*.^25^ where further information can be found. Note, minor modifications have been made, namely the removal of a hypothetical pathway and mitotic exit signal.

The full SAC model is taken from the literature^25^ with minor modifications and consists of 14 species and 21 interactions (Fig. 2). Its derivation and the meaning of its elements is described in detail by Henze *et al*.^25^. The unattached and attached kinetochore is modeled as molecular species “Kinetochore Unattached” (KinU) and “Kinetochore Attached” (KinA), respectively, which can be seen as the input of the checkpoint mechanism. KinU and KinA are discrete, counting the number of unattached and attached kinetochores, respectively, and thus ranging here from 0 to 92. As output, a high concentration of APC:MCC means that the checkpoint is active and thus the progression of the cell cycle is blocked (“wait”). A high concentration of APC/C:Cdc20 means a deactivation of the checkpoint thus allowing cell cycle progression (“go”). Therefore, the checkpoint mechanism is operating incorrectly if APC/C:Cdc20 appears while there is an unattached kinetochore left (i.e. “Unattached Kinetochore” species KinU is present).

#### Simulation of the full SAC model

The setup of the ReaDDY simulator is described in Methods Section 2.3 with the reactions corresponding to the full SAC model. Exact specifications of the parameters are provided in Table 3.

**Table 3:** Spatial parameters specifications of the Full SAC Model.

Species	Diffusion in *μm*^2^ *s*^−1^	Particles	Initial Concentration in *μM*
KinU	0.00	92	—
KinA	0.00	0	—
O-Mad2	16.61	409	0.15
C-Mad2	16.61	51	0.01875
Cdc20	12.97	600	0.22
BubR1:Bub3	7.92	354	0.13
APC/C	5.23	245	0.09

**Figure 3:**
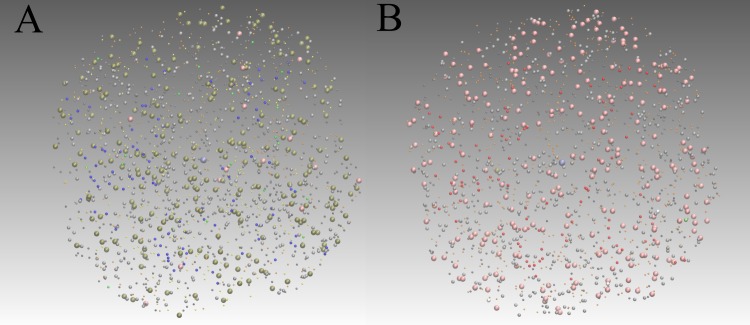
Stochastic simulation snapshots from the stochastic particle simulation of Model 1 using the efficient ReaDDy simulator^26^. Simulation time: 3 CPU days. Specification files can be found in the Methods Methods Section supp:readdy. (**A**) Initial state. All 92 kinetochores are unattached (small blue balls). (**B**) State after checkpoint deactivation. All kinetochores are attached (small red balls). Activated APC/C molecules are shown in pink.

**Figure 4:**
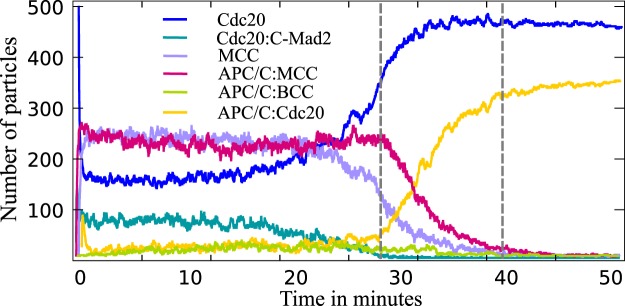
Concentration over time plot from the stochastic particle simulation of Model 1 showing the desired switching behavior. During metaphase (more than one kinetochore is unattached, until first dashed line) the level of APC/C:Cdc20 is low and APC/C:MCCs concentration is high. They switch quickly after the last kinetochore is properly attached (time between dashed lines).

Figure 3 shows snapshots from the detailed stochastic particle simulations of Model 1. The transition from unattached to attached kinetochores (blue balls to red balls) as well as the switch from inhibiting complexes to active ones (brownish balls to pink balls) can be seen. The quantitative switching can be also observed by plotting the species’ concentration over time (see Fig. 4), which we can use for comparing this simulation with coarse-grained models below.

#### Organizational analysis of the full SAC model (long timescale)

**Figure 5:**
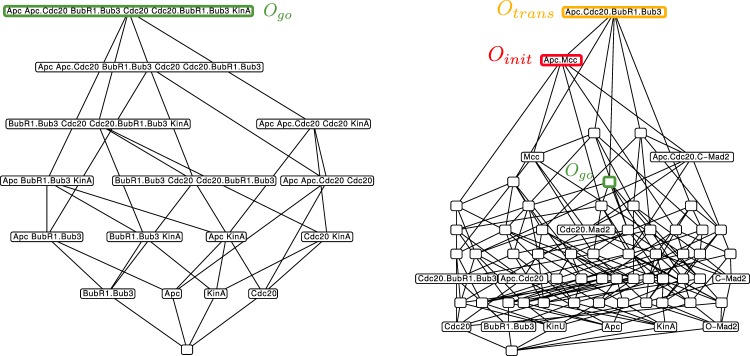
Hasse diagram of organizations from Model 1 in different modifications. Left: Organizational diagram of unmodified Model 1 (including attachment reaction KinU → KinA and the spontaneous decay of O-Mad2). The only interesting organization (see the condition in Sec. 3.1.2) is highlighted green and corresponds to all steady state species Right: Organizational diagram of modified Model 1 (no attachment reaction KinU → KinA or the spontaneous decay of O-Mad2). Here, only new species are depicted for the sake of clarity. Now there exist three interesting organizations, which are highlighted: *O*_*init*_ = {APC/C, APC/C:Cdc20, APC/C:Cdc20:C-Mad2, APC/C:MCC, BubR1:Bub3 C-Mad2 Cdc20 Cdc20:BubR1:Bub3, Cdc20:Mad2, KinU, MCC O-Mad2} corresponds to the species initially present (no attached kinetochores): *O*_*trans*_ = {APC/C, APC/C:Cdc20, APC/C:Cdc20:BubR1:Bub3, APC/C:Cdc20:C-Mad2, APC/C:MCC, BubR1:Bub3, C-Mad2, Cdc20, Cdc20:BubR1:Bub3, Cdc20:Mad2, KinA, KinU, MCC, O-Mad2} corresponds to the transitional phase (both attached and unattached kinetochores are present): *O*_*go*_ = {APC/C, APC/C:Cdc20, BubR1:Bub3, Cdc20, Cdc20:BubR1:Bub3, KinA, O-Mad2} is the same organization as on the left side (only attached kinetochores).

Model 1 has 16 organizations (see Fig. 5). From Theorem 1 of ref.^10^ we know that for every fixed point of the dynamical ODE model, the set of molecules with strictly positive concentrations in that fixed point is an organization of the reaction network (see also Methods Section). Thus, the organizations represent those subspaces of the state space in which the differential equation model can display stationary behavior or other attractors only. Note that there are 2^14^ − 16= 16368 other subspaces (i.e. combination of species) in which there cannot be any stationary behavior.

We can now further simplify the situation by asking which of these subspaces (organizations) are biologically meaningful. In order to do so we formulate domain knowledge as constraints. The following constraints represents the demand that a meaningful state should have a kinetochore, a Cdc20 protein and a BubR1:Bub3 protein complex in at least one form (i.e., bound or unbound).

Condition for an “interesting” organization for Model 1:

(KinA OR KinU) AND

(Cdc20 OR Cdc20:C-Mad2 OR MCC OR Cdc20:BubR1:Bub3 OR APC/C:Cdc20 OR APC/C:MCC OR

APC/C:Cdc20:C-Mad2 OR APC/C:Cdc20:BubR1:Bub3) AND

(BubR1:Bub3 OR MCC OR Cdc20:BubR1:Bub3 OR APC/C:MCC OR APC/C:Cdc20:BubR1:Bub3).

Using these constraints, only one organization remains. This means that, under biologically meaningful conditions (all proteins present as in the real cell), the system will reach one and only one organization (as desired) no matter what kind of kinetic rates and kinetic laws are chosen^29^.

#### Organizational analysis of the Full SAC Model in pro-meta phase (Model 1b, short timescale)

For investigating Model 1 at a short time scale during which no kinetochore attachment takes place, we remove the reaction “KinU → KinA” and the decay of O-Mad2, as it is relatively slow. The result is a much larger number of organizations (64) whose hierarchy is depicted by Fig. 5. The reason for this increased number is that we get more closed sets due to the removal of kinetochores attachment and also more self-maintaining sets due to the removal of the decay reaction O-Mad2, because it is now easier to maintain O-Mad2.

The lattice of organizations provides some insight into the dynamics of the model. For example, only the top organization contains the complex APC/C:Cdc20:BubR1:Bub3, thus this complex is not contained in any organization containing KinA or KinU alone. In other words, the complex APC/C:Cdc20:BubR1:Bub3 appears only during the transition, when both attached and unattached kinetochores are present, a fact that is difficult to see in the network model (Fig. 2). Note that the formation of APC/C:Cdc20:BubR1:Bub3 generates O-Mad2, which can sequester Cdc20, and thus could function as an additional force that keeps the checkpoint activated and that operates only during the transition phase.

The analysis shows that there are many subspaces with potential stationary behaviors (or attractors), thus a detailed analysis would be difficult or at least take some effort. Therefore, in the following, we will simplify the model by trying to conserve the organizations that matter and removing those organizations that are not relevant.

### Reduced SAC Model (Model 2)

The reduced SAC model (Model 2) is obtained from Model 1 by manually lumping several species into abstract species like “Promotor”, “Activator” and “Inhibitor”. As a result, the model reduces to 7 species and 8 reactions, making it more comprehensible, but still leaving a relatively large state space with a complex dynamics, as demonstrated in the following organizational analysis. The relation between species and organizations of the two models can be obtained from Table 4, mapping detailed species of Model 1 to abstract species of Model 2.

**Table 4:** Overview of the Model 2.

Abstract species (Model 2)	Detailed species (Model 1)	Remark
KinA	KinA	Kinetochore attached
KinU	KinU	Kinetochore unattached
Activator	Cdc20	Activator
Promotor	APC/C	(inactive) Promotor that can be activated.
Promotor_A	APC/C:Cdc20	Active Promotor, checkpoint deactivated, “go” signal
Inhibitor	O-Mad2, C-Mad2, C-Mad2:Cdc20, BubR1:Bub3, MCC	Inhibitor for the Promotor
Promotor_I	APC/C:MCC, APC/C:BCC, APC/C.C-Mad2:Cdc20	Inactive Promotor, inhibited such that it cannot be activated directly. “Wait” signal.

#### Simulation of the reduced SAC model

The setup of the ReaDDY simulator is described in Methods Section supp:readdy.with the reactions corresponding to the reduced SAC model. Exact specifications of the parameters are provided in Table 5.

**Table 5:** Spatial parameters specifications of the Reduced SAC Model.

Species	Diffusion in *μm*^2^ *s*^−1^	Particles	Initial Concentration in *μM*
KinU	0.00	92	—
KinA	0.00	0	—
Activator	12.97	5500	0.22
Inhibitor	7.92	3250	0.13
Promotor	5.23	2250	0.09

**Figure 6:**
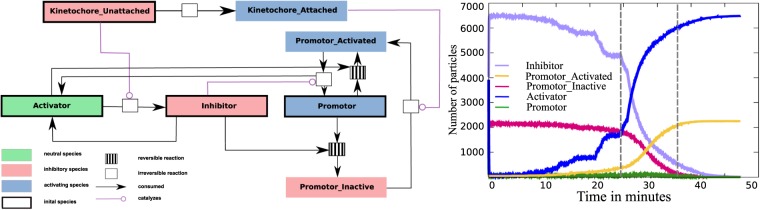
Left: Reaction network of the reduced SAC Model (Model 2). The meaning of symbols and colors is explained in the legend in the bottom left corner. Right: Concentration over time plot from the stochastic particle simulation of Model 2. During metaphase the level of “Activated Promotor” is low and “Inactivated Promotor concentration is high. They switch fast after the last kinetochore is properly attached (time between dashed lines).

The output of the ReaDDy simulation (see Fig. 6, Right) clearly shows the switch from inactive to active Promotor. A single kinetochore can maintain the SAC signal and only switches to its active form after the final attachment. The transition from Inhibitor to Activator takes place stepwise during the attachment process, as desired. This result indicates that Model 2 presents a valid coarse-graining of the full model, as it mimics the central SAC behavior.

#### Organizational analysis of the reduced SAC Model (long timescale)

**Figure 7:**
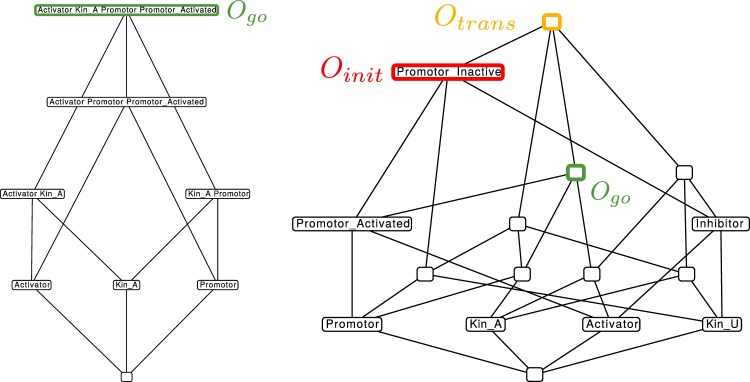
Hasse diagram of organizations for Model 2 in different modifications. Left: Organizational diagram of unmodified Model 2 (including attachment reaction KinU → KinA). The only interesting organization is highlighted in green. Right: Organizational diagram of modified Model 2 (no attachment reaction KinU → KinA). Only new species are shown for overviews sake. Again there exist three interesting organizations, which are highlighted. *O*_*init*_ = {Activator, Inhibitor, Promotor, Promotor_A, Promotor_I, KinU} corresponds to the initial present species (no attached kinetochores). *O*_*trans*_ = {Activator, Inhibitor, Promotor, Promotor_A, Promotor_I, KinU, KinA} corresponds to the transitional phase (both, attached and unattached kinetochores are present). *O*_*go*_ = {Activator, Promotor, Promotor_A, KinA} same organization as on the left side (only attached kinetochores).

Model 2 has 8 organizations (see Fig. 7, left), i.e., 8 organizations less than Model 1. For an organization to be “interesting”, we require that it contains a kinetochore, an Activator or Inhibitor, which can be bound to the Promotor, and a Promotor in any state.

Condition for an “interesting” organization for Model 2:

(KinA OR KinU) AND

(Activator OR Inhibitor) AND

(Promotor OR Promotor_A OR Promotor_I).

Again, there is only one organization, *O*_*go*_ = {Activator, KinA, Promotor, Promotor_A}, fulfilling this constraint (top organization in Fig. 7, left), which represents the inactivated checkpoint (“go”).

#### Organizational analysis of the reduced SAC Model in pro-meta phase (Model 2b, short timescale)

For studying the reduced model at a short time scale (before attachment), we again remove the attachment of kinetochores. Note that there is no need to remove O-Mad2 decay, since it does not appear here any more. The resulting lattice of organizations still contains 16 organizations, but only three are interesting with respect to the constraint above. They represent the initially activated checkpoint with all kinetochores unattached (only KinU but not KinA present) *O*_*init*_ = {Activator Inhibitor KinU Promotor Promotor_A Promotor_I}, the transitional states where attached and unattached kinetochores are present *O*_*trans*_ = *O*_*init*_ ∪ {KinA} (top organization in Fig. 7, right) and the final state with the checkpoint being inactivated *O*_*go*_.

### Coarsest SAC Model (Model 3)

**Figure 8:**
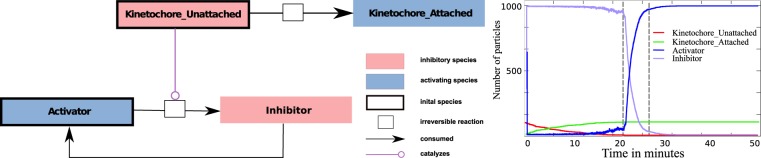
Left: Reaction network of the coarsest SAC Model (Model 3). The meaning of symbols and colors is explained in the legend in the bottom left corner. Right: Concentration over time plot from the stochastic particle simulation of Model 3. During metaphase the level of “Activator” is low and “Inhibitors” concentration is high. They switch quickly after the last kinetochore is properly attached (time between dashed lines).

We derive the coarsest SAC Model, based on the approximate aggregation method (explained in Section S1.5, such that a minimal set of reactions remains, displaying the set of interesting organizations. That is, the model should consider the attachment of kinetochores as well as the checkpoint activation and inhibition. Several detailed models of SAC are available, such as^25,41,42^, as well as manually constructed abstract models, such as^4,43–48^. Here, the coarsest model is derived automatically from Model 1 by applying the approximate aggregation. Applying this method to the full model results in the following network with only four species and three reactions (Fig. 8): KinU → KinA, Activator + KinU → KinU + Inhibitor, Inhibitor → Activator.

#### Simulation of the coarsest SAC model

The setup of the ReaDDY simulator is described in Methods Section 2.3 with the reactions corresponding to the reduced SAC model. Exact specifications of the parameters are provided in Table 6.

**Table 6:** Spatial parameters specifications of the Coarsest SAC Model.

Species	Diffusion in *μm*^2^ *s*^−1^	Particles	Initial Concentration in *μM*
KinU	0.00	92	—
KinA	0.00	0	—
Activator	12.97	1000	0.22
Inhibitor	11.42	0	0.00

The ReaDDy output (see Fig. 8, Right) shows the switch once more. However, now the transition is sharper, that is, the gradual increase of the Activator before the final attachment is less pronounced. The switch from Inhibitor to Activator only takes place after the last kinetochore is attached. This simple model explains the main SAC function but lacks behavioral details.

#### Organizational analysis of the coarsest SAC Model (long timescale)

**Figure 9:**
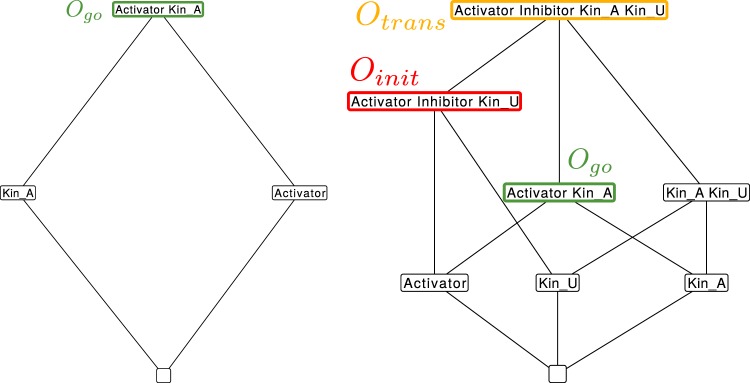
Hasse diagram of organizations from Model 3 in different modifications. Green boxes signal a ‘go’ state, red ones a ‘wait’ state and black ones are not of interest. Left: Organizational diagram of unmodified Model 3 (including attachment reaction KinU → KinA). Right: Organizational diagram of modified Model 3 (no attachment reaction KinU → KinA). There are three meaningful organizations (red, yellow and green boxes) that correspond to mitotic transition states, as desired, before attachment, during attachment and after attachment.

Model 3 has 4 organizations (see Fig. 9, left), thus 4 organizations less than Model 2. For an organization to be “interesting”, we require that it contains a kinetochore and an Activator or Inhibitor:

Condition for an “interesting” organization for Model 3:

(KinA OR KinU) AND

(Activator OR Inhibitor).

Again, there is only one “interesting” organization, *O*_*go*_ = {A, KinA}, which represents the desired mitotic exit state.

#### Organizational analysis of the coarsest SAC Model in pro-meta phase (Model 3b, short timescale)

For studying the transition dynamics in the coarsest model at a short time scale, we again remove the attachment of kinetochores, so that only the following two reactions remain for the organizational analysis: {Activator + KinU → KinU + Inhibitor, Inhibitor → Activator}.

The resulting lattice of organizations contains 8 organizations (8 less than Model 2), and, again, three organizations are interesting, with respect to the constraint above. Mitotic transition can now be explained as a transition between these three organizations, starting with the initially activated checkpoint with all kinetochores unattached *O*_*init*_ = {A, KinU}, over an intermediate period where attached and unattached kinetochores are present *O*_*trans*_ = {A, I, KinU, KinA}, and the final phase with the checkpoint being inactivated *O*_*go*_ = {A, KinA} (Fig. 9).

#### Stochastic state transition analysis of the coarsest SAC Model (long time scale)

In the following we perform a detailed study of the dynamics of this coarsest model by various techniques: PRISM model checking, hybrid ODE-stochastic simulation and spatial stochastic simulations. All these simulations are able to reconstruct the switching behavior as in the detailed model.

**Figure 10:**
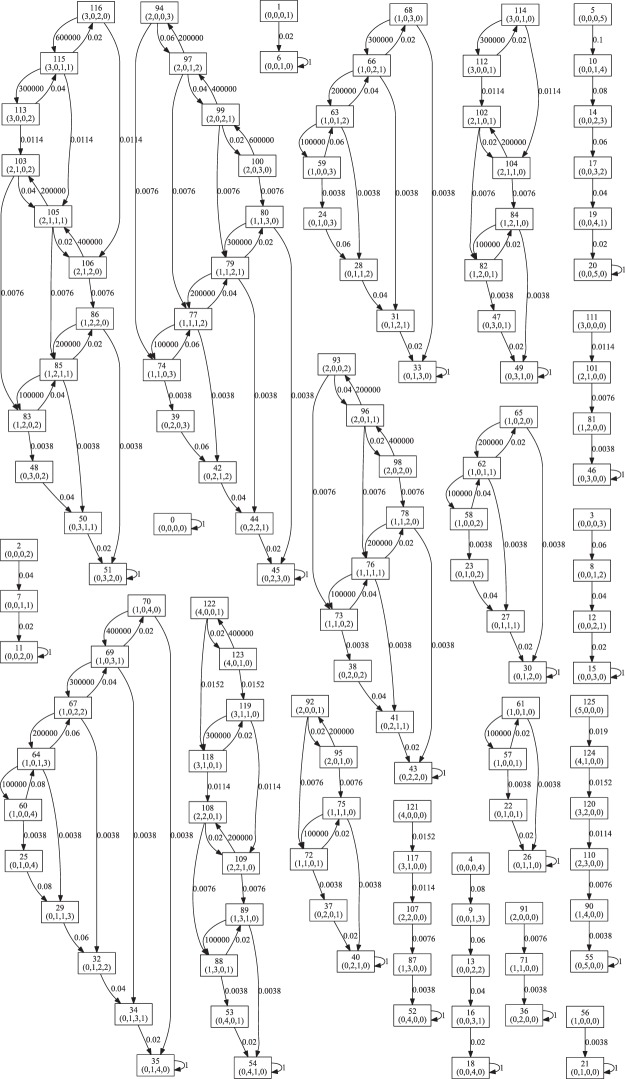
State transition graph of the CTMC model for Model 3 generated by PRISM. State labels show index and population count, e.g., 62 (1,0,1,1) denotes that there are 1 KinU, 0 KinA, 1 A and 1 I in state 62. Arrows denote the transitions between states, numbers on the arrows denote the rates of the transitions.

The PRISM-based stochastic state transition analysis^32^ of Model 3 (long time scale) confirms that the organizations for this example are: {KinA, A}, {A}, {KinA}, {} and that there are no other discrete organizations. The expected leaving time of them are all infinite (all good SCCs are bottom SCCs; see Sec. 2.6 and^32^ for more details), no transitions between any of them are possible. For illustration, Fig. S1 presents the state transition graph with a maximum of 5 molecules (see also Fig. 10).

**Figure 11:**
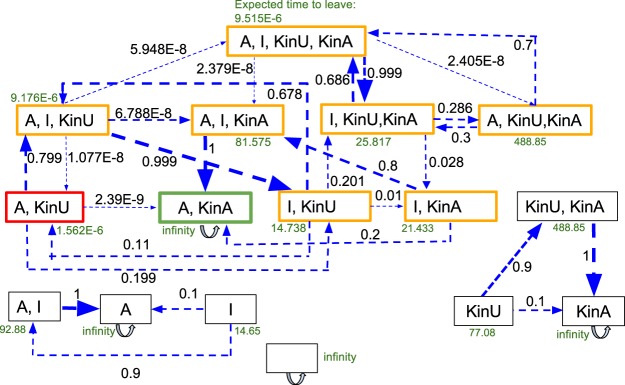
State transition analysis of unmodified Model 3 assuming a maximum of 10 molecules (including attachment reaction KinU → KinA). We show all set of states that the system can be in (2^4^ = 16 set states, whereby the actual state space is 1,001, depending on the distribution of the 10 molecules). The green state refers to the checkpoint inactivation (“go”), the red one to the initial state and the yellow ones to intermediate states. States depicted in black cannot be reached in our model and are not interesting, following the criteria in Sec. 3.1.2. Depending on the reaction rates of the model, the estimated time to leave a state is depicted in green, where an infinite time means that this state is never left. The blue arrows indicate the propensity to go into the state the arrow is pointing at (black numbers correlate with the regarding arrows thickness). Those numbers can be seen as likelihood of the state transition and are calculated exactly based on the model. See^32^ for method details.

Looking at organizations only, we study in more detail the transient stochastic dynamics using the PRISM model checking framework^32^. Figure 11 presents the transition probabilities between (internal generators of) organizations, and the expected time to leave each of them, which confirms the desired behavior. Here, we can clearly see how the system undergoes a transition from the short term organizations (modified Model 3) to the long term organization {A, KinA} of Model 3, representing checkpoint inactivation (“go”). Starting with {I, KinU}, the Activator appears only very seldomly (i.e. the transition to {A, I, KinU} is unlikely). Then attached kinetochores appear (likely transition to {I, KinU, KinA}. If an Activator now appears (i.e., transition {I, A, KinU, KinA}), the Activator quickly vanishes again (going back to {I, KinU, KinA} with high probability). As soon as no kinetochore is still attached, Activator molecules become more likely (likely transition to {A,I, KinA}), from which the final “go” state is likely reached ({A, KinA}, in which no Inhibitor is present.

#### Stochastic state transition analysis of the coarsest SAC Model in pro-meta phase (Model 3b, short time scale)

The PRISM-based stochastic state transition analysis (following^32^) shows that at a short time scale, each of the “interesting” organizations (red and green boxes in Fig. 9) are stable, which can be shown by a full state-space analysis using PRISM^32^. When limiting the total number of molecules to N_max = 5 we get the state transition diagram presented in Fig. S1.

The analysis shows that the discrete organizations for this example are the same as the above graph. The expected leaving times of them are all infinite, that is, there are no transitions between any of them (all good SCCs are bottom SCCs; see Sec. 2.6 and^32^ for more details).

### Discrete 4-state model (Model 4)

**Figure 12:**
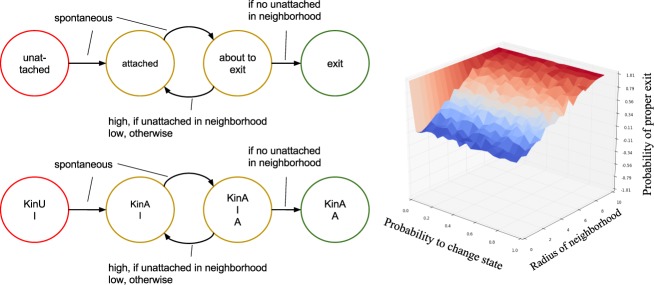
Discrete state Model 4. Left: State transition diagram of a single compartment. The initial state for all compartments is ‘unattached’. The cell has failed if one compartment is in state ‘exit’ while another one is in state ‘unattached’ at the same time. Presented below is the same model with references to species from Model 3. Right: Simulation of the discrete 4 state model using Python. Here, we show the probability of checkpoint success, that is, while one kinetochore is still ‘unattached’ no other one is in the ‘exit’ state. The x-axis (0–1) shows the probability p of a transition from ‘attached’ to ‘about to exit’ while the y-axis (0–10) shows the distance radius of a neighborhood relation. The graphic shows that the rate of success is independent of the probability p and only depends on the range of a neighborhood. Namely, the more neighbors are considered before moving on to ‘exit’ state the more likely the mitosis is successful.

In order to get a spatial model that can be analyzed by model checking we coarse-grain further and derive a discrete stochastic model in which each compartment (representing a kinetochore) has only four states (see Fig. 12, Left).

In the following we derive the four-state model from Model 3. where each state represents a combination of species that are present (concentration > 0) in a compartment with a single kinetochore. Note that in this situation KinU and KinA cannot appear at the same time. Thus we obtain six possible states (see Table 7).

**Table 7:** Deviation of the four-state model (Model 4) via six states. Two states can be eliminated, because they are unlikely due to a quick production of the Inhibitor.

Outputsignal	KinU	KinA	Activator	Inhibitor	State
‘Wait’	x			x	‘unattached’
‘Wait’	x		x	x	Unlikely, because Activator is quickly converted to Inhibitor
‘Go’	x		x		Unlikely, because Activator is quickly converted to Inhibitor
‘Wait’		x		x	‘attached’
‘Wait’		x	x	x	‘about to exit’
‘Go’		x	x		‘exit’

By assuming that the activation of the Inhibitor by the unattached kinetochore (KinU + Activator → KinU + Inhibitor) is fast, two states become unlikely, thus we arrive at a 4-state model. The connection between the 4-state model and Model 3 is shown in the left panel of Fig. 12.

Transitions from ‘unattached’ to ‘attached’ states and to ‘about to exit’ happen spontaneous. Furthermore, we assume that state transitions within one compartment are influenced by its neighbors. For more details on the model, see Sec. S1.2.

#### Simulation of the 4-state model

The outcome of the simulation is shown in the right panel of Fig. 12. We show the probability of a SAC success with varying transition rates from the ‘attached’ state to the ‘about to exit’ state and neighbor relations. A dysfunction occurs in the system if one node is ‘unattached’ while another one is already in ‘exit state’.

**Figure 13:**
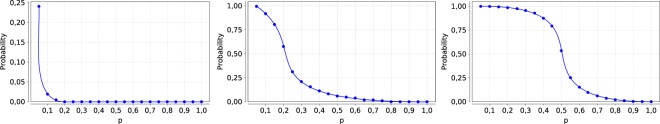
Outcome of the simulated model Model 4 with PRISM. Only 80 of the 92 kinetochores could be realized. The x-axis presents the transition probability with which state ‘attached’ goes to ‘about to exit’ and the y-axis the probability of a successful SAC. Left: This example shows lined up kinetochores, meaning 2 neighbors for each kinetochore. Middle: A more connected graph with five neighbors per kinetochore. Right: An even more connected graph with 7 neighbors per kinetochore. This shows that the success depends slightly on the aforementioned probability but considerably more on the neighborhood range. Clearly a graph that is not fully connected results in a checkpoint defect.

The 3D-plot shows clearly that the SAC only works to 100% if all nodes are considered neighbors or the rate to change spontaneously to ‘about to exit’ is zero. This is independent of the distribution of the kinetochores (positioned randomly or in a straight line; see Fig. 13). This shows that the SAC signal is not local, but indeed has to function global and that one kinetochore is sufficient to maintain the SAC signal.

#### PRISM stochastic model checking of the four-state model

The coarse-grained stochastic 4-state model now allows us to apply probabilistic model checking with PRISM in order to get exact probabilities (and not only experimentally obtained ones like before). These results (see Fig. 13) also confirm the validity of the model and show how space would counteract the functioning of the checkpoint mechanism. From this it follows that space should not play a role in checkpoint function. In other words, a global neighborhood must be assumed and local signaling travel times should not matter.

#### Spatial organization analysis of the four-state model

**Figure 14:**
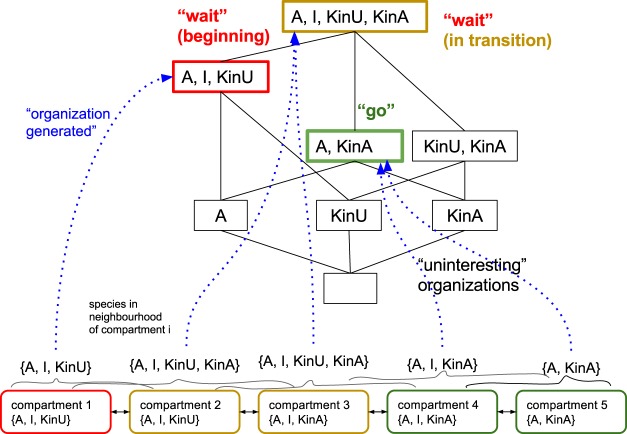
Illustration of how spatial organizations are related to the lattice of organizations. The spatial organization of a compartment is defined as the organization generated by the species found in its neighborhood (incl. the compartment). For example, the species in the neighborhood of compartment 4 are A, I, KinA and the organization generated by it {A, KinA}. Note that compartment 4 has the same set of species as compartment 3, but the set of species in its neighborhood is A, I, KinA and thus different from that of compartment 3.

Finally, we show in the following how the coarse-graining concepts described above can be applied to space, by identifying local chemical organizations. For this, we consider space as a set of compartments interconnected by diffusion and transport processes (Fig. 14, bottom). Each compartment holds a well-stirred chemical system, that is, a compartment’s state is described by a concentration vector. We further assume that each compartment holds one kinetochore, thus KinA and KinU cannot be contained in the same compartment at the same time (see Fig. 14, bottom, for an example situation).

Following the idea of spatial chemical organizations^29^ we need to define a neighborhood. Here, assuming a neighborhood of radius 1, i.e. the neighborhood of compartment 3 includes itself plus compartments 2 and 4. The spatial organization of a spatial location (here, a compartment id) is defined by the organization generated by the molecules contained in the neighborhood of this location^29^. Given a set of molecules, we *generate* an organization by first adding to the set all possible reaction products until no new molecule can be added any more. Then we remove those molecules that are not sufficiently produced until we reach a closed and self-maintaining set of species (see Methods and ref.^29^).

As we can see in Fig. 14, two compartments containing the same molecular species (e.g., A, I, KinA) can be mapped to different organizations, which depends on a compartment’s neighborhood. The local organization generated can be interpreted as an upper bound on the compartment’s fate in the time-scale defined implicitly by the neighborhood and rate of transport and diffusion processes, which provides a fundamentally different (coarse-grained) view on the system than by looking at the state space only.

## Conclusion

Starting from a detailed particle-based simulation model, which takes days of simulation time for a single trajectory, we have shown various coarse-grainings leading to models that can be executed in seconds or studied completely by exact model checking methods in minutes. Our novel methods (applied here in combination, and introduced elsewhere previously) have shown to be useful, i.e. allowing a complex model to be coarse-grained to models that can be executed efficiently.

From a biological perspective we could confirm that communication between kinetochores has to be fast, i.e., global. Local communication would reduce the reliability of the checkpoint. Thus the spatial location of kinetochores is not important and models need not to take the spatial locations of kinetochores into account.

From a general modeling perspective, we conclude that having a rich toolbox of coarse-graining techniques at hand can be useful for: (1) simulating and analyzing complex models more efficiently; and (2) gaining a better understanding of the mechanisms that explain the observed behavior. In order to obtain coarse-grained models that are also meaningful, automatic methods must (still) be complemented by domain-expert knowledge. Ideally, this knowledge can also be formalized, for example, as constraints as shown above.

From a methodological point of view, there are many research pathways leading further from here, such as the development of a spatial organization-oriented coarse graining framework sketched above or the inclusion of semantic technologies like controlled vocabularies^49^ to link different coarse-graining levels.

## Supplementary information

Supplementary Information
