# Supplementary material for: Multi-scale stochastic organization-oriented coarse-graining exemplified on the human mitotic checkpoint
Source: Sci Rep. 2019 Mar 7;9:3902. doi: 10.1038/s41598-019-40648-w (PMC6405958; doi:10.1038/s41598-019-40648-w)
Supplement: Supplementary file 1 — Supplementary Information [file 41598_2019_40648_MOESM1_ESM.pdf]

Multi-scale Stochastic Organization-Oriented Coarse-Graining Exemplified on the Human Mitotic Checkpoint

– Supplementary Material –

Richard Henze[1,a], Chunyan Mu[2,a], Mate Puljiz[3], Nishanthan Kamaleson[4], Jan Huwald[1], John Haslegrave[6], Pietro Speroni di Fenizio[1], David Parker[4], Christopher Good[5], Jonathan E. Rowe[4], Bashar Ibrahim[7,*], Peter Dittrich[1,*]

[1]Faculty of Mathematics and Computer Science, Friedrich Schiller University, Germany
[2]School of Computing, Teesside University, UK
[3]Faculty of Electrical Engineering and Computing, University of Zagreb, Croatia
[4]School of Computer Science, University of Birmingham, UK
[5]School of Mathematics, University of Birmingham, UK
[6]Mathematics Institute, University of Warwick, UK
[7]Chair of Bioinformatics, Matthias Schleiden Institute, Friedrich Schiller University of Jena, Germany
[a]these authors contributed equally to this work
[*]corresponding bashar.ibrahim@uni-jena.de or peter.dittrich@uni-jena.de

Contents

1 Reaction Network Models (Model 1 - Model 3) 2
2 Supplementary Figure S1: State Transition Graph of a CTMC Model 3b) 4
3 Method: Approximate Aggregation Method for Model Reduction 5
3.1 Relation of this method to other model reduction techniques 6
References 7

1 Reaction Network Models (Model 1 - Model 3)

Model 1 as rea-file format.
For the modified "short timescale" version (Model1b) we remove two reactions: "KinU -> KinA" and "O-Mad2 ->". The modified version represents a short timescale at which no kinetochore gets attached and O-Mad2 does not decay.

```
# Number of Components
14
# Components
KinA
KinU
Apc
Mcc
Apc.Mcc
BubR1.Bub3
Cdc20
Apc.Cdc20
Cdc20.Mad2
C-Mad2
O-Mad2
Cdc20.BubR1.Bub3
Apc.Cdc20.C-Mad2
Apc.Cdc20.BubR1.Bub3
# Number of Reactions
21
# Reactions
1.0 KinU -> 1.0 KinA
1.0 Apc 1.0 Mcc -> 1.0 Apc.Mcc
1.0 Apc.Mcc -> 1.0 Apc 1.0 Mcc
1.0 Apc 1.0 Cdc20 -> 1.0 Apc.Cdc20
1.0 KinU 1.0 O-Mad2 -> 1.0 KinU 1.0 C-Mad2
1.0 Cdc20.Mad2 1.0 BubR1.Bub3 -> 1.0 Mcc
1.0 Mcc -> 1.0 Cdc20.Mad2 1.0 BubR1.Bub3
1.0 Cdc20 1.0 C-Mad2 -> 1.0 Cdc20.Mad2
1.0 C-Mad2 -> 1.0 O-Mad2
1.0 Cdc20.Mad2 -> 1.0 Cdc20 1.0 O-Mad2
1.0 Apc.Cdc20 1.0 Mcc -> 1.0 Apc 1.0 Cdc20 1.0 Mcc
1.0 Cdc20 1.0 BubR1.Bub3 -> 1.0 Cdc20.BubR1.Bub3
1.0 Cdc20.BubR1.Bub3 -> 1.0 Cdc20 1.0 BubR1.Bub3
1.0 Apc.Cdc20 1.0 C-Mad2 -> 1.0 Apc.Cdc20.C-Mad2
1.0 Apc.Cdc20.C-Mad2 -> 1.0 Apc.Cdc20 1.0 C-Mad2
1.0 Apc.Cdc20.C-Mad2 1.0 BubR1.Bub3 -> 1.0 Apc.Mcc
1.0 Apc.Mcc -> 1.0 Apc.Cdc20.C-Mad2 1.0 BubR1.Bub3
1.0 Apc.Mcc 1.0 KinA ->1.0 Apc.Cdc20.BubR1.Bub3 1.0 O-Mad2 1.0 KinA
1.0 Apc.Cdc20.BubR1.Bub3 -> 1.0 Apc.Cdc20 1.0 BubR1.Bub3
1.0 O-Mad2 ->
1.0 Mcc 1.0 KinA -> 1.0 Cdc20.BubR1.Bub3 1.0 O-Mad2 1.0 KinA
```

Model 2 as rea-file format.
For the modified “short timescale” version (Model2b) we remove reaction: “KinU -> KinA”.

```
# Number of Components
7
# Components
Kin_A
Kin_U
Activator
Promotor
Promotor_Activated
Inhibitor
Promotor_Inactive
# Number of Reactions
9
# Reactions
1.0 Kin_U -> 1.0 Kin_A
1.0 Inhibitor -> 1.0 Activator
1.0 Activator 1.0 Kin_U -> 1.0 Inhibitor 1.0 Kin_U
1.0 Promotor_Inactive 1.0 Kin_A -> 1.0 Promotor_Activated 1.0 Kin_A
1.0 Promotor 1.0 Activator -> 1.0 Promotor_Activated
1.0 Promotor_Activated 1.0 Inhibitor -> 1.0 Promotor 1.0 Activator 1.0 Inhibitor
1.0 Promotor 1.0 Inhibitor -> 1.0 Promotor_Inactive
1.0 Promotor_Inactive -> 1.0 Promotor 1.0 Inhibitor
1.0 Promotor_Activated -> 1.0 Promotor 1.0 Activator
```

Model 3 as rea-file format.
For the modified “short timescale” version (Model3b) we remove reaction: “KinU -> KinA”.

```
# Number of Components
4
# Components
Activator
Inhibitor
Kin_A
Kin_U
# Number of Reactions
3
# Reactions
1.0 Kin_U -> 1.0 Kin_A
1.0 Activator 1.0 Kin_U -> 1.0 Inhibitor 1.0 Kin_U
1.0 Inhibitor -> 1.0 Activator
```

2 Supplementary Figure S1: State Transition Graph of a CTMC Model 3b)

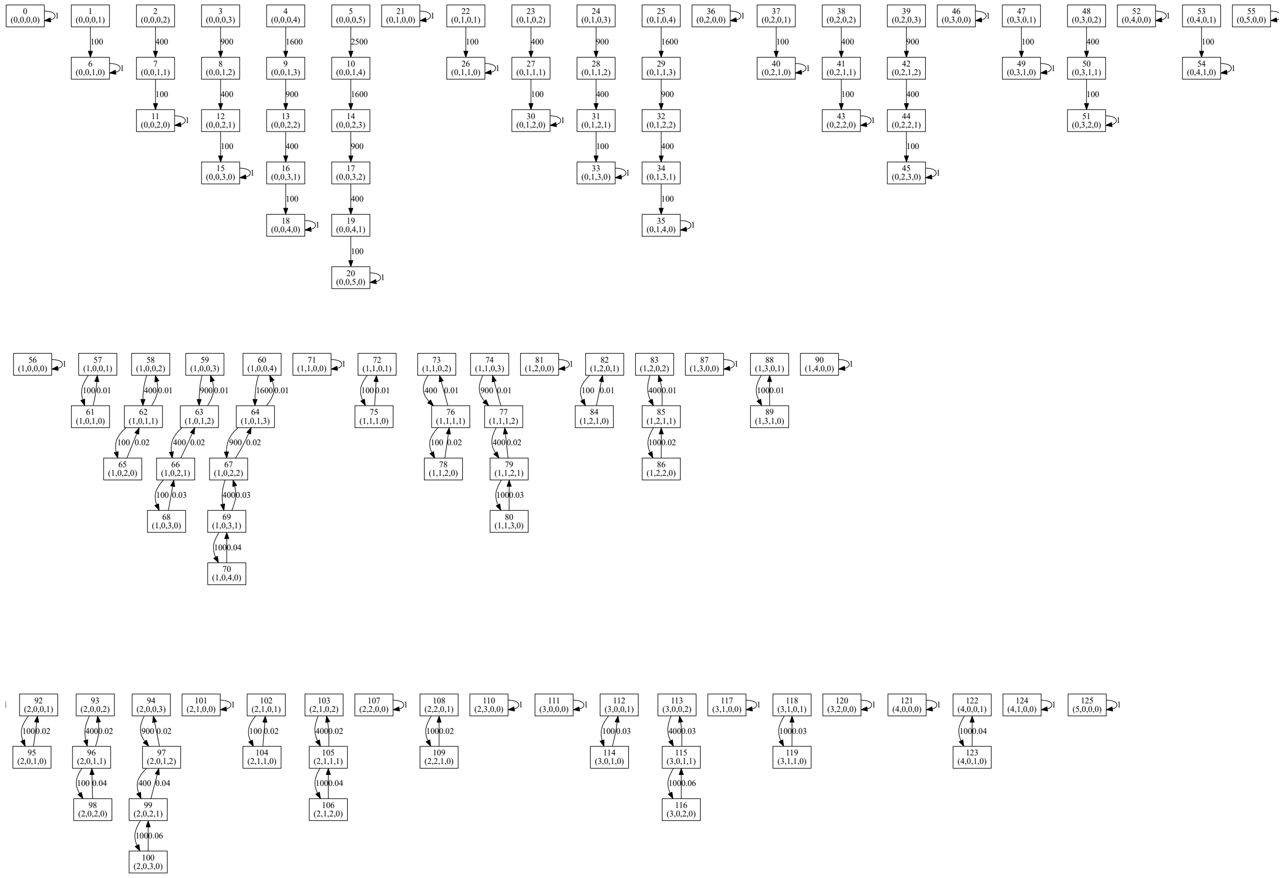

Figure 1. State transition graph of the CTMC model for Model3b generated by PRISM. State labels show index and population count, e.g., 24 (0,1,0,3) denotes that there are 0 KinU, 1 KinA, 0 A and 3 I in state 24. Arrows denote the transitions between states, numbers over the arrows denote the rate of the transition.

3 Method: Approximate Aggregation Method for Model Reduction

In this section we recall a method[24] for finding approximate aggregations of an ODE system $\dot{x} = A(x)$ where $A\colon \mathbb{R}^n \to \mathbb{R}^n$ is a quadratic polynomial. This case is important as any set of interesting biochemical reaction will usually incorporate rules for producing one type of particles by combining two different types of reactants. This kind of dynamics translates to A being a polynomial map of degree two. Oddly enough, reactions involving terms of order 3 and higher are rarely encountered as they can, at least conceptually, be simulated as successive reactions of order two.

Firstly, recall that finding aggregations of the system $\dot{x} = A(x)$ is equivalent to finding aggregations of the map $x \mapsto A(x)$[43]. It can be checked that any quadratic polynomial $A\colon \mathbb{R}^n \to \mathbb{R}^n$ can be written as

$$A(x) = A(0) + DA(0)x + \tfrac{1}{2}(DA(x) - DA(0))x$$

where DA denotes the differential of A. Note that the second order term in the equation above is indeed linear as the other power of x is hidden within the differential. It is possible to write our function in this convenient form only because we assumed that $A(x)$ is a quadratic polynomial and this sort of argument does not straightforwardly generalize to polynomials of higher degrees. Since entries of DA are affine maps we can further write

$$DA(x) = DA(0) + \sum_{i=1}^{n} x_i(DA(e_i) - DA(0))$$

where e_i for $1 \le i \le n$ are unit vectors of the canonical basis in $\mathbb{R}^n$ and x is a vector with entries $x = (x_1, \ldots, x_n)$. Thus,

$$A(x) = A(0) + DA(0)x + \tfrac{1}{2}\sum_{i=1}^{n} x_i(DA(e_i) - DA(0))x$$

and we see that vector $A(x)$ can be written as a linear combination of vectors $A(0)$, $DA(0)x$, $DA(e_1)x$, $\ldots$, $DA(e_n)x$. We will now show that any aggregation which is simultaneously compatible with matrices $DA(0)$, $DA(e_1)$, $\ldots$, $DA(e_n)$ must also be compatible with A. To see this, let us denote by Ξ the coarse graining matrix associated to a particular aggregation which is simultaneously compatible with matrices $DA(0)$, $DA(e_1)$, $\ldots$, $DA(e_n)$. This means that we can find matrices $\widehat{DA(0)}$, $\widehat{DA(e_1)}$, $\ldots$, $\widehat{DA(e_n)}$ for which the following equations hold:

$$\Xi DA(0) = \widehat{DA(0)}\Xi, \qquad \Xi DA(e_1) = \widehat{DA(e_1)}\Xi, \qquad \ldots, \qquad \Xi DA(e_n) = \widehat{DA(e_n)}\Xi.$$

Then applying Ξ to $A(x)$ we get:

$$\begin{aligned}\Xi A(x) &= \Xi A(0) + \Xi DA(0)x + \tfrac{1}{2}\sum_{i=1}^{n} x_i(\Xi DA(e_i) - \Xi DA(0))x \\ &= \Xi A(0) + +\widehat{DA(0)}\Xi x + \tfrac{1}{2}\sum_{i=1}^{n} x_i(\widehat{DA(e_i)} - \widehat{DA(0)})\Xi x\end{aligned}$$

where the last expression is clearly a function of Ξx which further means that Ξ is compatible with map $A(x)$.

The converse to this fact is also known to be true[45]. Together, this allows us to reduce our problem of finding aggregations for A to that of finding aggregations simultaneously compatible with each of the matrices $DA(0)$, $DA(e_1)$, $\ldots$, $DA(e_n)$.

In fact, rather than looking for aggregations compatible simultaneously with $DA(0)$, $DA(e_1)$, $\ldots$, $DA(e_n)$ which may not even exist, it seems more reasonable to look for a set of matrices $\widetilde{DA(0)}$, $\widetilde{DA(e_1)}$, $\ldots$, $\widetilde{DA(e_n)}$ which approximate them in some matrix norm while at the same time being simultaneously compatible with a (preferably large) set of aggregations S. The first step in doing so would be to do this for just one n-by-n matrix M.

Recall that an m-by-n ($m \le n$) 0-1 matrix Ξ with exactly one entry equal to 1 in each column is called an aggregation matrix and these are in 1-1 correspondence with the partitions of the set $\{1, 2, \ldots, n\}$. For each such Ξ let V_Ξ be the set of all n-by-n matrices coarse grained by Ξ. One can show that this set is a linear subspace of the set of all matrices of order n which we denote by $M_n(\mathbb{R})$[24]. Thus, after fixing some matrix norm, one can orthogonally project matrix M onto V_Ξ in order to obtain the best approximation $\widetilde{M}$ which is coarse grained by Ξ.

But this only ensures that $\widetilde{M}$ is coarse grained by Ξ, whereas one would like to get as many coarse-grainings as possible. Note however that if Ξ_1 and Ξ_2 are two aggregation matrices, then $V_{\Xi_1} \cap V_{\Xi_2}$ is again a linear subspace of $M_n(\mathbb{R})$ consisting precisely of matrices which are coarse grained by both Ξ_1 and Ξ_2. Projecting orthogonally onto this subspace will yield an approximation of M which has at least those two valid reductions.

This idea extends to an arbitrary number of aggregation matrices, and one is inclined to ask how large a subset of aggregation matrices $\{\Xi_1, \ldots, \Xi_r\} \subseteq \{\Xi_\mathfrak{p} \mid \mathfrak{p} \text{ is a partition of } \{1, 2, \ldots, n\}\}$ can be while ensuring that the distance from A to its orthogonal projection onto $V_{\Xi_1} \cap \cdots \cap V_{\Xi_r}$ is kept within a given error threshold. Note that the number of partitions of

a set $\{1,\ldots,n\}$ is already super-exponential in n, and going through all subsets of those would yield a growth rate greater than doubly exponential, thus making any brute force approach ineffective.

Instead, in Algorithm 1 below we propose running through all aggregations and retaining in a set S only those for which induced projections produce an approximation matrix within the error threshold ε. The final approximation is then obtained by projecting onto $\bigcap_{\mathfrak{p}\in S} V_{\Xi_\mathfrak{p}}$. The reasoning behind this is that a matrix M that is ε-close to a subspace $V \cap W$ cannot be further than ε from either V or W. This however is not a guarantee that the converse, which is needed here, is true.

Algorithm 1 Finding a nearby matrix that can be coarse grained

Input: (n-by-n matrix) M
(error threshold) $\varepsilon > 0$

$S = \{\}$
for partition $\mathfrak{p}$ of $\{1,2,\ldots,n\}$ **do**
  $M' =$ projection of M onto $V_{\Xi_\mathfrak{p}}$
  if $\|M - M'\| < \varepsilon$ **then**
    add $\mathfrak{p}$ to S
  end if
end for
$V = \bigcap_{\mathfrak{p}\in S} V_{\Xi_\mathfrak{p}}$
$\tilde{M} =$ projection of M onto V

Output: (the approximate matrix) $\tilde{M}$
(the set of aggregations) S

It is not hard to modify Algorithm 1 so that it works with a set of matrices. We give the modification below as Algorithm 2. Again, there is no guarantee that the resulting matrices are ε close to the initial ones, but the same argument as before justifies this approach.

Algorithm 2 Finding approximate matrices that can be jointly coarse grained

Input: (n-by-n matrices) $M_1,\ldots,M_k$
(error threshold) $\varepsilon > 0$

$S = \{\}$
for partition $\mathfrak{p}$ of $\{1,2,\ldots,n\}$ **do**
  $M_i' =$ projection of M_i onto $V_{\Xi_\mathfrak{p}}$
  if $\|M_i - M_i'\| < \varepsilon$, for all $1 \le i \le k$ **then**
    add $\mathfrak{p}$ to S
  end if
end for
$V = \bigcap_{\mathfrak{p}\in S} V_{\Xi_\mathfrak{p}}$
$\tilde{M}_i =$ projection of M_i onto V

Output: (the approximate matrices) $\tilde{M}_1,\ldots,\tilde{M}_k$
(the set of aggregations) S

Going back to our original problem, assume that we used this algorithm to obtain matrices $\widetilde{DA(0)}$, $\widetilde{DA(e_1)}$, ..., $\widetilde{DA(e_n)}$ and the set of aggregations S that simultaneously coarse grains each of them. Then the approximate quadratic map $\widetilde{A}(x)$ is simply given by

$$\widetilde{A}(x) = A(0) + \widetilde{DA(0)}x + \tfrac{1}{2}\sum_{i=1}^{n} x_i(\widetilde{DA(e_i)} - \widetilde{DA(0)})x$$

and the ODE system $\dot{x} = \widetilde{A}(x)$ can be aggregated using any of the coarse grainings from the set S.

3.1 Relation of this method to other model reduction techniques

The problem that standard model reduction techniques are attempting to solve usually consists of finding the evolution of a quantity of interest whose dynamics is modeled by a high-dimensional ODE system. The goal is then to find a low-dimensional model in which the evolution of the said quantity will match its actual evolution as accurately as possible.[46,47]

In our approach we focus our attention to the high dimensional model itself and from there we attempt to derive quantities whose evolution can be *exactly* computed using reduced models. The guiding idea here being that it should be possible to automatically derive certain "conservation laws" that govern the dynamics of our system. The goal of our

approach is learning a hierarchical structure of the system under consideration and seeing how its organizational structure fits together. The model reduction part then comes as a by-product of this process.

Consequently, the approximate aggregation method discussed above favors models with rich structure, models with abundance of coarse grainings. This is perhaps best seen on an extremely simple example. The following two-dimensional linear ODE model is a modification of an example by Rowe et al.[44]

$$\begin{pmatrix} \dot{x} \\ \dot{y} \end{pmatrix} = \begin{pmatrix} .01 & .99 \\ .98 & .02 \end{pmatrix} \begin{pmatrix} x \\ y \end{pmatrix}$$

It can be calculated that both singular values of this matrix are of magnitude close to 1. The classical model reduction techniques based on SVD decomposition would therefore fail to reduce this model since both singular values are non-negligible and cutting off the smaller one would lead to a vastly different model.

$$\begin{pmatrix} \dot{x} \\ \dot{y} \end{pmatrix} = \begin{pmatrix} .472 & .659 \\ .342 & .477 \end{pmatrix} \begin{pmatrix} x \\ y \end{pmatrix}$$

Our method on the contrary produces a system close to the original

$$\begin{pmatrix} \dot{x} \\ \dot{y} \end{pmatrix} = \begin{pmatrix} .015 & .985 \\ .985 & .015 \end{pmatrix} \begin{pmatrix} x \\ y \end{pmatrix}$$

but in which variables x and y can be aggregated together to give a reduced model

$$\dot{z} = z$$

where $z = x + y$.

References

1. Morgan, D. O. *The Cell Cycle: Principles of Control (Primers in Biology) (Primers in Biology)* (Sinauer Associates, Inc., 2006).
2. Szallasi, Z., Stelling, J. & Periwal, V. *System modeling in cellular biology* (MIT Press, Cambridge, MA, 2006).
3. Karr, J. R. *et al.* A whole-cell computational model predicts phenotype from genotype. *Cell* **150**, 389–401 (2012).
4. Doncic, A., Ben-Jacob, E. & Barkai, N. Evaluating putative mechanisms of the mitotic spindle checkpoint. *Proceedings of the National Academy of Sciences of the United States of America* **102**, 6332–6337 (2005).
5. Mochida, S., Rata, S., Hino, H., Nagai, T. & Novák, B. Two bistable switches govern m phase entry. *Current Biology* **26**, 3361–3367 (2016).
6. Cardelli, L. Morphisms of reaction networks that couple structure to function. *BMC systems biology* **8**, 84 (2014).
7. Johnson, T., Bartol, T., Sejnowski, T. & Mjolsness, E. Model reduction for stochastic camkii reaction kinetics in synapses by graph-constrained correlation dynamics. *Physical biology* **12**, 045005 (2015).
8. Feret, J., Danos, V., Krivine, J., Harmer, R. & Fontana, W. Internal coarse-graining of molecular systems. *Proceedings of the National Academy of Sciences* **106**, 6453–6458 (2009).
9. Henkel, R. *et al.* Notions of similarity for systems biology models. *Briefings in bioinformatics* bbw090 (2016).
10. Dittrich, P. & Speroni di Fenizio, P. Chemical organization theory. *Bull. Math. Biol.* **69**, 1199–1231 (2007).
11. Musacchio, A. & Salmon, E. D. The spindle-assembly checkpoint in space and time. *Nat. Rev. Mol. Cell Biol.* **8**, 379–393 (2007).
12. Ibrahim, B. Toward a systems-level view of mitotic checkpoints. *Prog. Biophys. Mol. Biol.* **117**, 217–224 (2015).
13. Manic, G., Corradi, F., Sistigu, A., Siteni, S. & Vitale, I. Molecular Regulation of the Spindle Assembly Checkpoint by Kinases and Phosphatases. *Int Rev Cell Mol Biol* **328**, 105–161 (2017).
14. Diogo, V., Teixeira, J., Silva, P. M. & Bousbaa, H. Spindle Assembly Checkpoint as a Potential Target in Colorectal Cancer: Current Status and Future Perspectives. *Clin Colorectal Cancer* **16**, 1–8 (2017).
15. Ibrahim, B. & Henze, R. Active transport can greatly enhance cdc20: Mad2 formation. *International journal of molecular sciences* **15**, 19074–19091 (2014).
16. Ibrahim, B., Schmitt, E., Dittrich, P. & Diekmann, S. In silico study of kinetochore control, amplification, and inhibition effects in mcc assembly. *BioSystems* **95**, 35–50 (2009).
17. Ibrahim, B. Systems Biology Modeling of Five Pathways for Regulation and Potent Inhibition of the Anaphase-Promoting Complex (APC/C): Pivotal Roles for MCC and BubR1. *OMICS* **19**, 294–305 (2015).

18. Henze, R., Dittrich, P. & Ibrahim, B. A dynamical model for activating and silencing the mitotic checkpoint. *Scientific Reports* **7** (2017).

19. Schoneberg, J. & Noe, F. ReaDDy–a software for particle-based reaction-diffusion dynamics in crowded cellular environments. *PLoS ONE* **8**, e74261 (2013).

20. Mu, C., Dittrich, P., Parker, D. & Rowe, J. E. Organisation-oriented coarse graining and refinement of stochastic reaction networks. *IEEE/ACM Transactions on Computational Biology and Bioinformatics* (2018).

21. Kreyssig, P. *et al.* Effects of small particle numbers on long-term behaviour in discrete biochemical systems. *Bioinformatics* **30**, 475–481 (2014).

22. Speroni di Fenizio, P. & Dittrich, P. Chemical Organizations at Different Spatial Scales. *Advances in Artificial Life* 1–11 (2007).

23. Kreyssig, P. *et al.* Cycles and the qualitative evolution of chemical systems. *PLoS ONE* **7**, e45772 (2012).

24. Puljiz, M. *On coarse graining and other fine problems*. Ph.D. thesis, University of Birmingham (2017).

25. Mu, C., Dittrich, P., Parker, D. & Rowe, J. E. Formal quantitative analysis of reaction networks using chemical organisation theory. In *Computational Methods in Systems Biology - 14th International Conference, CMSB 2016, Cambridge, UK, September 21-23, 2016, Proceedings*, 232–251 (2016).

26. Kamaleson, N., Parker, D. & Rowe, J. E. Finite-horizon bisimulation minimisation for probabilistic systems. In *Proc. 2016 International Symposium on Model Checking of Software (SPIN'16)*, vol. 9641 of *LNCS*, 147–164 (Springer, 2016).

27. Ibrahim, B., Diekmann, S., Schmitt, E. & Dittrich, P. In-silico modeling of the mitotic spindle assembly checkpoint. *PLoS ONE* **3** (2008).

28. Ibrahim, B. In silico spatial simulations reveal that MCC formation and excess BubR1 are required for tight inhibition of the anaphase-promoting complex. *Mol Biosyst* **11**, 2867–2877 (2015).

29. Gross, F., Bonaiuti, P., Hauf, S. & Ciliberto, A. Implications of alternative routes to APC/C inhibition by the mitotic checkpoint complex. *PLoS Comput. Biol.* **14**, e1006449 (2018).

30. Ibrahim, B. A mathematical framework for kinetochore-driven activation feedback in the mitotic checkpoint. *Bulletin of mathematical biology* **79**, 1183–1200 (2017).

31. Ibrahim, B., Dittrich, P., Diekmann, S. & Schmitt, E. Stochastic effects in a compartmental model for mitotic checkpoint regulation. *Journal of Integrative Bioinformatics* **4** (2007).

32. Ibrahim, B., Dittrich, P., Diekmann, S. & Schmitt, E. Mad2 binding is not sufficient for complete cdc20 sequestering in mitotic transition control (an in silico study). *Biophysical Chemistry* **134**, 93–100 (2008).

33. Ibrahim, B. Mathematical analysis and modeling of DNA segregation mechanisms. *Math Biosci Eng* **15**, 429–440 (2018).

34. Verdugo, A., Vinod, P. K., Tyson, J. J. & Novak, B. Molecular mechanisms creating bistable switches at cell cycle transitions. *Open biology* **3**, 120179 (2013).

35. Knupfer, C., Beckstein, C., Dittrich, P. & Le Novere, N. Structure, function, and behaviour of computational models in systems biology. *BMC Syst Biol* **7**, 43 (2013).

36. Benkö, G. *et al.* A topological approach to chemical organizations. *Artificial Life* **15**, 71–88 (2009).

37. Fontana, W. Algorithmic chemistry. Tech. Rep., Los Alamos National Lab., NM (USA) (1990).

38. Fontana, W. & Buss, L. W. "the arrival of the fittest": Toward a theory of biological organization. *Bulletin of Mathematical Biology* **56**, 1–64 (1994).

39. di Fenizio, P. S., Dittrich, P., Banzhaf, W. & Ziegler, J. Towards a theory of organizations. In *German Workshop on Artificial Life (GWAL 2000), in print, Bayreuth*, vol. 5, 2000 (2000).

40. Peter, S. & Dittrich, P. On the Relation between Organizations and Limit Sets in Chemical Reaction Systems. *Advances in Complex Systems* **14**, 77–96 (2011).

41. Kwiatkowska, M., Norman, G. & Parker, D. PRISM 4.0: Verification of probabilistic real-time systems. In *Proc. CAV'11*, vol. 6806 of *LNCS* (2011).

42. Kleinberg, J. & Tardos, E. *Algorithm Design* (Addison–Wesley, Boston, 2006).

43. Jacobi, M. N. Hierarchical organization in smooth dynamical systems. *Artif. Life* **11**, 493–512 (2005).

44. Rowe, J. E., Vose, M. & Wright, A. H. State aggregation and population dynamics in linear systems. *Artif. Life* **11**, 473–492 (2005).

45. Rowe, J. E., Vose, M. & Wright, A. H. Differentiable coarse graining. *Theoret. Comput. Sci.* **361**, 111–129 (2006).

46. Gorban, A. N., Kazantzis, N. K., Kevrekidis, I. G., Öttinger, H. C. & Theodoropoulos, C. *Model reduction and coarse-graining approaches for multiscale phenomena* (Springer, Berlin, 2006).

47. Benner, P., Gugercin, S. & Willcox, K. A survey of projection-based model reduction methods for parametric dynamical systems. *SIAM review* **57**, 483–531 (2015).
